# Experimental evolution with *E. coli *in diverse resource environments. I. Fluctuating environments promote divergence of replicate populations

**DOI:** 10.1186/1471-2148-10-11

**Published:** 2010-01-13

**Authors:** Tim F Cooper, Richard E Lenski

**Affiliations:** 1 Department of Biology and Biochemistry, University of Houston, Houston, Texas 77204, USA; 2 Department of Microbiology and Molecular Genetics, Michigan State University, East Lansing, Michigan 48824, USA

## Abstract

**Background:**

Environmental conditions affect the topology of the adaptive landscape and thus the trajectories followed by evolving populations. For example, a heterogeneous environment might lead to a more rugged adaptive landscape, making it more likely that replicate populations would evolve toward distinct adaptive peaks, relative to a uniform environment. To date, the influence of environmental variability on evolutionary dynamics has received relatively little experimental study.

**Results:**

We report findings from an experiment designed to test the effects of environmental variability on the adaptation and divergence of replicate populations of *E. coli*. A total of 42 populations evolved for 2000 generations in 7 environmental regimes that differed in the number, identity, and presentation of the limiting resource. Regimes were organized in two sets, having the sugars glucose and maltose singly and in combination, or glucose and lactose singly and in combination. Combinations of sugars were presented either simultaneously or as temporally fluctuating resource regimes. This design allowed us to compare the effects of resource identity and presentation on the evolutionary trajectories followed by replicate populations. After 2000 generations, the fitness of all populations had increased relative to the common ancestor, but to different extents. Populations evolved in glucose improved the least, whereas populations evolving in maltose or lactose increased the most in their respective sets. Among-population divergence also differed across regimes, with variation higher in those groups that evolved in fluctuating environments than in those that faced constant resource regimens. This divergence under the fluctuating conditions increased between 1000 and 2000 generations, consistent with replicate populations evolving toward distinct adaptive peaks.

**Conclusions:**

These results support the hypothesis that environmental heterogeneity can give rise to more rugged adaptive landscapes, which in turn promote evolutionary diversification. These results also demonstrate that this effect depends on the form of environmental heterogeneity, with greater divergence when the pairs of resources fluctuated temporally rather than being presented simultaneously.

## Background

An understanding of the factors that influence the reproducibility, and therefore the predictability, of adaptation is of fundamental importance to any evolutionary theory of biological diversity. For example, divergence among populations can be caused by adaptation to different environments, or by chance differences in evolutionary history such as mutational order and drift, which may constrain subsequent evolution and promote divergence even when populations evolve in and adapt to the same environment [1-5]. Traditionally, these processes were studied by comparing extant populations and attempting to correlate organismal traits with environmental parameters to infer the action of selection [6,7]. Experimental studies complement the comparative approach by allowing evolutionary dynamics to be monitored in real time, including controlled tests to examine the effects of environmental conditions on evolutionary processes and outcomes [reviewed in [8,9]].

In recent years, a number of evolution experiments have been performed with microbes to examine the extent of divergence among replicate populations, often finding evidence of striking parallel evolution at phenotypic and even genetic levels [10-22]. However, most of these studies compared populations adapting to constant and uniform environments. Hence, the effects of environmental heterogeneity, an important factor in the evolution of natural populations, were not addressed. Those studies that included heterogeneity have tended to focus on its effect on within-population dynamics, particularly on how it influences the evolution of specialist and generalist phenotypes [13,14,19,23-30]. How environmental heterogeneity affects among-population dynamics, in particular the reproducibility of evolutionary trajectories, has received only limited attention [but see [14,15,31]].

Wright's metaphor of an adaptive landscape provides a framework for considering the adaptation and divergence of populations [5,32]. This landscape is typically visualized as a two-dimensional representation of the highly multi-dimensional genetic space, onto which a fitness surface is projected. The relative height of this surface represents the fitness of an organism with a particular genotype. Natural selection, then, typically pushes populations toward peaks with high fitness. In the simplest case, the adaptive landscape has only one fitness peak, such that replicate populations should eventually converge on the same solution (although the time required for convergence may be exceedingly long). However, if interactions exist between mutations, such that the fitness effect of any one mutation depends on whether or not another one is already present (i.e., mutations interact epistatically), then the landscape may be more rugged, with multiple peaks separated by valleys [5,32-34]. In this case, several different local peaks might be accessible to a population starting at a given point in genetic space, depending on the order in which beneficial mutations arise that escape loss by random drift. Natural selection inhibits movement of populations between peaks separated by intermediate types having lower fitness; therefore, rugged landscapes increase the probability that replicate populations will diverge toward distinct adaptive peaks.

Besides the intrinsic interactions between mutations, a key extrinsic factor that has been suggested to influence the ruggedness of an adaptive landscape is environmental heterogeneity [13-15,35,36]. For example, if there are genetic trade-offs in the ability of an organism to adapt simultaneously to two resources, then temporal fluctuations between the resources might create a composite landscape having more fitness peaks than were present in either single resource environment, which would then promote divergence among populations [35,36]. In a related vein, Wright's model of "mass selection under changing conditions" invokes temporal changes in the environment as a process that can facilitate the movement of evolving populations between different fitness peaks [37,38].

Here, we aim to extend previous evolution experiments by considering the effect of environmental heterogeneity on the adaptation and divergence of replicate populations, where that heterogeneity reflects both the number (either one or two) and the presentation (simultaneous or fluctuating) of resources. To this end, we propagated 42 populations of *Escherichia coli *for 2000 generations in 7 defined environments differing in resource identity, number, and presentation. After this time, the fitness of each evolved population was measured relative to the common ancestor in several environments, and statistical analyses were performed to determine the effect of the selective environment on the extent of adaptation and degree of fitness divergence among replicate populations.

## Results

### Direct fitness response in different environments

Table 1 shows the average change in relative fitness over the six replicate populations that evolved under each environmental regime for the full 2000 generations. Fitness increased significantly for all treatment groups (Table 1), and indeed for all of the individual populations within each group (data not shown), thus demonstrating that the populations had adapted to each of the selective environments.

**Table 1:** Final mean fitness of groups evolved under each resource regime for 2000 generations.

Group	Grand Mean (± SEM)	P*
glu&mal set		
glu	1.210 (± 0.009)	**<0.001^†^**
mal	1.266 (± 0.013)	**<0.001**
glu/mal	1.218 (± 0.022)	**<0.001**
glu+mal	1.246 (± 0.017)	**<0.001**
		
glu&lac set		
glu^‡^	1.210 (± 0.009)	**<0.001**
lac	1.426 (± 0.027)	**<0.001**
glu/lac	1.294 (± 0.033)	**<0.001**
glu+lac	1.303 (± 0.018)	**<0.001**

*;One-tailed *t*-test. The null hypothesis is that the mean fitness equals 1.0. SEM values are based on the 6 replicate populations in each treatment group, except for the mal group which had only 5 surviving replicates.

^†^Values that remain significant (*P *< 0.05) following sequential Bonferroni correction for the four tests performed within each resource set are shown in bold.

^‡^The glu group is repeated for clarity.

Next, we sought to assess whether the average fitness gains differed among the groups evolved in the different environments. To do this, we performed one-way ANOVAs for the glu&mal and glu&lac selection sets, nesting population under the resource environment. This analysis showed that populations that evolved under the different environments varied in the extent to which their fitness had increased (glu&mal *F_3,175 _*= 7.62, *P *< 0.001; glu&lac *F_3,183 _*= 52.14, *P *< 0.001). We then performed pairwise comparisons between the treatment groups within each of the two sets (Table 2). In five of the twelve pairwise comparisons, the difference in the average fitness gains between groups was statistically significant after applying a sequential Bonferroni correction. The glu group had the smallest improvement of all groups. The mal and lac groups had larger direct fitness responses than the groups evolved in the corresponding mixed environments containing the same resources, although this difference was only significant for the lac group. Fitness increases between alternating and simultaneous presentations of the two resource combinations were not significantly different in either the mal or lac set of environments. It is also interesting to note that in comparisons between the two resource sets, the groups in the glu&lac set tended to have larger fitness increases than the corresponding groups in the glu&mal set (difference in relative fitness and two-tailed *t*-test: lac vs. mal 0.160, *P *< 0.001; glu/lac vs. glu/mal 0.076, *P *= 0.087; glu+lac vs. glu+mal 0.057, *P *= 0.040).

**Table 2:** Comparison of direct fitness responses between different groups.

Comparison	Difference in direct response	P*
glu&mal set		
glu vs. mal	-0.056	**0.006^†^**
glu vs. glu/mal	-0.008	0.748
glu vs. glu+mal	-0.036	0.085
mal vs. glu/mal	0.048	0.114
mal vs. glu+mal	0.020	0.400
glu+mal vs. glu/mal	0.028	0.330
		
glu&lac set		
glu vs. lac	-0.216	**<0.001**
glu vs. glu/lac	-0.084	0.036
glu vs. glu+lac	-0.094	**<0.001**
lac vs. glu/lac	0.132	**0.012**
lac vs. glu+lac	0.122	**0.003**
glu+lac vs. glu/lac	0.010	0.807

*Two-tailed *t*-test.

^†^Values that remain significant (*P *< 0.05) following sequential Bonferroni correction for the six comparisons performed within each resource set are shown in bold.

### Extent of divergence among replicate populations

To address whether the replicate populations within each treatment group were evolving toward the same or different adaptive peaks, we first tested whether they had diverged significantly from one another in terms of their fitness levels in the environment in which they had evolved. Significant divergence in their direct responses would indicate that the replicate populations had followed different trajectories and would be consistent with them evolving toward different adaptive peaks. For each group, an ANOVA was performed to test the significance of the among-population variance in fitness in their selection environment after 2000 generations. As shown in Table 3, the among-population variation was significant for the glu/mal, lac and glu/lac groups, with the glu/mal and glu/lac groups both remaining significant even after accounting for multiple tests.

**Table 3:** Among-population variation in direct response after 2000 generations.

Group	Mean fitness	*F*	P*
glu&mal set			
glu	1.210	1.031	0.411
mal	1.269	1.459	0.235
glu/mal	1.218	15.347	**<0.001**^†^
glu+mal	1.246	1.913	0.110
			
glu&lac set			
glu^‡^	1.210	1.031	0.411
lac	1.423	2.669	0.035
glu/lac	1.294	17.64	**<0.001**
glu+lac	1.303	1.646	0.167

*Significance of population effect determined by one-way ANOVA.

^†^Values that remain significant (*P *< 0.05) following sequential Bonferroni correction for the four tests performed within each resource set are shown in bold.

^‡^The glu group is repeated for clarity.

### Variation in fitness in alternative environments

**Figure 1:**
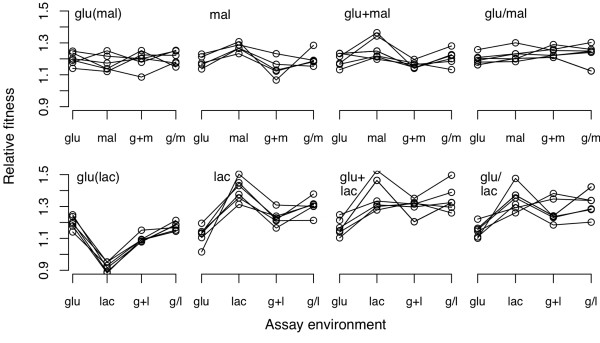
**Direct and correlated fitness responses of the replicate populations in each group**. Panels labelled glu(mal) and glu(lac) correspond to the same glucose-evolved populations but assayed in resource environments comprising the glu&mal or glu&lac sets, respectively. Each point is the mean of five fitness assays. Lines connect the same population across the different environments.

The preceding results indicate that significant among-population divergence evolved in at least two of the seven treatment groups. However, this assessment is based on only one trait, namely fitness in the particular environment in which each group of populations evolved. Therefore, we cannot exclude the possibility that populations in the other groups were also evolving toward different adaptive solutions but had similar direct fitness responses. It might be possible to differentiate populations that show similar direct responses by their correlated responses to other environments [39-42]. We therefore measured the fitness of each population in all of the environments making up each section of the experiment; for example, the fitness values of populations in the mal group were measured in the glu, mal, glu/mal and glu+mal environments. The results of all these assays are summarized in Figure 1. Net declines in fitness were not observed as correlated responses, at least across the range of environments tested, with one conspicuous exception: all six populations that evolved with glucose as the sole resource lost fitness when they were tested in the lactose-only environment. Interestingly, the reciprocal tradeoffs were not observed. All six lactose-evolved populations were more fit in lactose than in glucose, but none was significantly less fit than the ancestor in the lactose-only environment. The absence of tradeoffs in the two-resource regimes is less surprising, however, since any mutation that was beneficial for exploiting one resource but detrimental with respect to the second resource would have been disadvantaged relative to any other beneficial mutation accessible that did not impose such a maladaptive pleiotropic effect.

We then performed two-way ANOVAs to determine whether there were significant (i) main effects of replicate populations across the environments, and (ii) interaction effects between replicate populations and environments (Table 4). The glu+mal group exhibited a significant population effect, while the lac, glu/lac and glu+lac groups all showed significant population-by-environment interactions; the interaction variation in the glu/lac group was significant even after a Bonferroni correction for multiple tests.

**Table 4:** Summary of analyses of variance for fitness of the replicate populations in each group across all environments.

	Source of variation			
	
	Population*		Population × environment*	
**Group**	***F***	***P***	***F***	***P***

glu&mal set				
glu	1.585	0.224	1.486	0.126
mal	1.273	0.334	1.590	0.113
glu/mal	2.168	0.112	1.380	0.176
glu+mal	4.086	0.015	0.413	0.972
				
glu&lac set				
glu	1.255	0.333	0.752	0.725
lac	0.996	0.453	1.891	0.035
glu/lac	1.384	0.285	3.327	**<0.001**^†^
glu+lac	2.186	0.110	2.018	0.022

*Significance of population and population-by-environment interaction effects determined by two-way ANOVA, with population analyzed as a random effect and environment as a fixed effect.

^†^Values that remain significant (*P *< 0.05) following sequential Bonferroni correction for the four tests performed within each resource set are shown in bold.

### Change in population divergence over time

Taking all of the direct and correlated responses together, there were three cases of significant variation among replicate populations after adjusting for multiple tests of the same hypothesis within each resource set. All three cases involved the glu&mal and glu&lac treatment groups that experienced temporally fluctuating environments, even though those represented only two of the seven total treatment groups. These results are consistent, therefore, with the hypothesis that fluctuating environments promote evolutionary divergence. However, it is also possible that the populations are converging on the same peak but are doing so at different speeds or by different routes. For example, transient divergence might be caused by stochastic differences in the order and timing of adaptations in replicate populations that would eventually converge to the same adaptive peak [16,43]. To evaluate this possibility, we examined whether the among-population variation in fitness in the selection environment was increasing or diminishing over time. Sustained or even increasing divergence would support the hypothesis that replicate populations were approaching different fitness peaks, whereas declining variation would tend to support transient divergence leading to eventual convergence [16,44].

**Figure 2:**
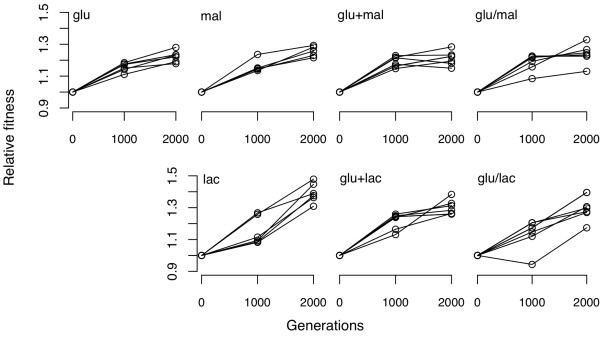
**Direct fitness responses of replicate populations within each group after 1000 and 2000 generations of experimental evolution**. All fitness values are expressed relative to the common ancestor. Each point is the mean of four assays performed in the same experimental blocks. Lines connect the same populations measured at 1000 and 2000 generations.

To that end, we calculated the variance component for fitness for each group's direct response at both generations 1000 and 2000 (using only those assays that were part of the same experimental blocks). Taking the square root of the variance component, √Var *W*, gives the standard deviation for fitness due to genetic factors (as opposed to measurement error) among the populations in each group, and it scales in a manner comparable to relative fitness. The estimated genetic variance in fitness among populations increased between 1000 and 2000 generations in the glu/mal group but not in the glu/lac group (Table 5). This variance component also increased in the glu group, but not in any of the other groups that experienced temporally constant regimes of either single or mixed resources. As is evident in Figure 2, the variance in the glu/lac group at generation 1000 was very strongly influenced by one population for which no measurable fitness gain had yet occurred. If we exclude that outlier population, then the variance component also increased between 1000 and 2000 generations for the glu/lac group (Table 5). These trends, while certainly not definitive proof, are thus consistent with the hypothesis that the replicate populations in the temporally fluctuating resource environments were evolving toward distinct fitness peaks.

**Table 5:** Temporal trend in the fitness variation among replicate populations, measured in the selective environment.

Group	1000 generations √Var *W*	2000 generations* √Var *W*	Δ √Var *W*
glu&mal set			
glu	0.013	0.025	0.013
mal	0.015	0.000^†^	-0.015
glu/mal	0.022	0.039	0.018
glu+mal	0.035	0.005	-0.030
			
glu&lac set			
glu^‡^	0.013	0.025	0.013
lac	0.072	0.025	-0.047
glu/lac	0.086	0.062	-0.024
glu/lac: omitting outlier population	0.012	0.048	0.036
glu+lac	0.036	0.031	-0.005

*The 2000-generation means and variances were calculated using only the 4 fitness assays that were performed in the same experimental blocks as the 1000-generation assays.

^†^Reported as zero because the calculated variance component was negative, whereas the real value must be zero or positive.

^‡^The glu group is repeated for clarity.

## Discussion

Since its introduction over seventy years ago, Wright's metaphor of the adaptive landscape has become one of the most influential concepts in evolutionary biology, yet empirical understanding of the structures of actual landscapes remains elusive. In this study, we monitored the evolution of 42 experimental populations of *E. coli *under 7 resource regimes for 2000 generations in order to investigate the effects of environmental complexity on their dynamics. We were interested, in particular, in whether heterogeneous resource environments would influence the repeatability of evolution by impacting the ruggedness of the adaptive landscape. Our results can be summarized as follows. (1) Populations evolved under all experimental regimes exhibited significant increases in fitness. (2) The magnitude of their fitness gains varied across the regimes, as did their correlated fitness responses to other regimes. (3) Among-population genetic variation for fitness was highest, and most sustained over time, in those groups that had evolved under fluctuating resource regimes. Below we discuss these findings in more detail.

### Extent and specificity of adaptation

After 2000 generations, the mean fitness of all populations had increased significantly under all seven resource regimes (Table 1). Prior to all fitness assays, ancestral and evolved competitors were both physiologically pre-conditioned in the competition environment. Therefore, the fitness gains of the evolved populations are the result of heritable adaptation rather than physiological acclimation. Each population had the opportunity to adapt to one of seven different resource regimes, but other aspects of their environment (e.g., temperature and base minimal medium) were common to all of the selection regimes. If genetic adaptation had occurred only in response to those shared aspects of the environment, then the magnitude of fitness gains would have been indistinguishable among the treatment groups. In fact, however, there was considerable variation in the average fitness improvement across the groups, ranging from ~21% in the glu group to ~43% for the lac group, with many of the pairwise comparisons between groups being significant (Table 2). These results demonstrate that a portion of the overall adaptation was specific to the particular resource regimen under which the populations had evolved.

### Effect of environment on patterns of divergence

We only found evidence for highly significant divergence among the replicate populations that evolved in the fluctuating resource environments, glu/mal and glu/lac (Table 3). To address whether this divergence was sustained over time, we also quantified the change in the variance for fitness between 1,000 and 2,000 generations (Table 5). Divergence among the replicate populations in the glu/mal group increased over the second half of the experiment, whereas divergence among the populations in the glu/lac group decreased over this period. However, the variation in the glu/lac group at 1,000 generations was strongly influenced by a single population that showed no fitness gain to that point. When this atypical population was excluded from the analysis, divergence trended higher in this group as well. On the whole, these results indicate that divergence of replicate populations was both significant and increasing over time only in those two groups that evolved in the temporally fluctuating environments. This finding is consistent with the hypothesis that the replicate populations in these treatment groups were evolving toward different adaptive peaks.

In principle, replicate populations could diverge from one another not only by selection acting on different beneficial mutations but also by drift and hitchhiking. Divergence by drift could occur through the accumulation of mutations that are neutral in the selective environment, but which might have some fitness effects in other environments. Deleterious mutations might hitchhike to high frequency if they become linked with a beneficial mutation [45,46], which could occur since the bacteria in our experiments are strictly asexual (i.e., they lack any mechanism for horizontal gene transfer). However, the *E. coli *strain we used has a very low total genomic mutation rate [47], which should limit the rates of substitution by drift and hitchhiking. Indeed, high-coverage whole-genome sequencing of another population founded from the same strain found that only three synonymous mutations achieved detectable frequencies in 20,000 generations [48]. Moreover, the patterns of correlated responses in 12 populations, again founded from the same ancestral strain, indicate that pleiotropic effects of beneficial mutations have been more important than mutation accumulation by drift or hitchhiking in explaining patterns of phenotypic evolution over 20,000 generations [10-12]. Therefore, it appears unlikely that drift or hitchhiking have contributed much, if at all, to the among-population divergence in our 2000-generation experiment, nor is it evident why any such effects would be stronger in the fluctuating environment treatments than in other treatments that experienced the same resources alone or in combination.

Selection can also lead to among-population divergence due to stochastic differences in the timing and order of beneficial mutations [43]. If only a single local adaptive peak is available, then this divergence should be transient. However, if mutations interact such that some mutations are beneficial only in combination with other mutations, then this 'sign epistasis' may generate multiple peaks separated by valleys of maladapted intermediates [33,49]. In the latter case, differences in the order in which the mutations arise could cause initially identical populations to evolve to different peaks, resulting in sustained divergence [33,49,50]. While stochastic differences in the timing and order of mutations might well contribute to population divergence, they cannot alone explain why the extent of variation and its persistence over time should differ between groups. Although we cannot rule out the possibility that the divergence in those groups that evolved with fluctuating resources might eventually prove to be transient (say, over tens of thousands of generations), this possibility would nevertheless imply differences in the structure of fitness landscapes between the various regimes that contributed to their different early dynamics of divergence. In future work, we intend to identify the genetic bases of the adaptation of these populations and then combine mutations to test directly for the presence of sign-epistatic interactions.

A possible caveat to this interpretation is that, because the benefit of adaptive mutations must be averaged across two different environments, those populations evolved in fluctuating environments may have experienced weaker effective selection than the populations that evolved in constant environments [51]. If so, then adaptive mutations would take longer to fix and the period of transient divergence would be extended. However, we did not observe substantially smaller fitness gains for the groups that evolved in the fluctuating environments (Table 1).

The greater divergence among populations in the fluctuating environments is consistent with the hypothesis that environmental heterogeneity can increase the ruggedness of the adaptive landscape and thereby increase the likelihood that replicate populations will find distinct fitness peaks [13-15,35,36]. However, it is less obvious why divergence was more pronounced in regimes with two resources that fluctuated temporally, as opposed to regimes that presented the same two resources simultaneously. Temporal fluctuations would presumably have favoured generalists that are well adapted to each of the alternating resources, whereas coexisting specialists might evolve when the resources are simultaneously present. That distinction, while important, still begs the question of whether independently evolved populations would achieve similar or diverse generalist types or admixtures of specialists under those respective scenarios. Although the specific adaptations that occurred in the populations are not yet known, we can imagine a scenario where one might well expect greater among-population divergence under the fluctuating resource regimes. Consider a mutation that increases fitness in the glucose component of a fluctuating environment, and which becomes fixed in one population. Despite conferring a net fitness advantage, this mutation might nevertheless reduce fitness in the lactose component of the environment. (In fact, such trade-offs are evident in most or all the populations that evolved in glucose, as seen in Figure 1). This deleterious side-effect will select for compensatory mutations that alleviate the fitness trade-off in the lactose environment [37,52-55]. Such compensatory mutations are beneficial only in combination with the previous mutation, and they would not evolve without the adaptation to glucose. As a consequence, the spectrum of potential future adaptations will differ from those available to a population that by chance fixed an alternative mutation that did not engender a trade-off, and therefore would not select for a compensatory mutation. By contrast, in those environments where two resources were presented simultaneously, an evolving lineage might, at least in principle, split into two subpopulations that each adapt to one resource while forgoing adaptation to the other. The two subpopulations might then be on different peaks, yet if all of the replicate lineages split into subpopulations in the same way, then the replicate populations per se might not diverge from one another.

A caveat to this conceptual distinction between simultaneous and fluctuating environments is the fact that *E. coli *typically uses multiple resources (including glucose and either lactose or maltose) sequentially rather than simultaneously [56-58]. Because only one resource is used at a time, the effect is to partition the environment physiologically, thus giving rise to two sequential adaptive landscapes just as in the fluctuating environment. However, in contrast to the externally imposed fluctuating regimes, the physiological separation between these successive landscapes is under genetic control. Preliminary results (T.F.C., unpublished data) indicate that four of the six glu+mal populations and all six of the glu+lac populations evolved some change in the activity of catabolite repression (whereby the presence of glucose represses the use of other sugars), in which case the resources may have been used simultaneously. These changes, if confirmed, might have reduced the among-population variation in those regimes where resources were presented simultaneously rather than sequentially.

Finally, a limitation of our analyses of the evolved lines thus far is that they only address variation in fitness. Thus, we cannot exclude the possibility that replicate populations might have diverged genetically, and even physiologically, in ways that nevertheless yield similar fitness levels in the assayed environments. Some of the potential targets of selection in this experiment include the length of the lag phase following transfer to fresh medium, maximum growth rate and, in mixed resource environments, regulation of substrate utilization preferences. Different combinations of changes in these traits might produce similar fitness gains [59,60]. Although measurements of additional characters can only increase the likelihood of detecting variation among populations, we see no obvious reason why adding traits would substantially affect the relative among-population variation across the different treatment groups.

By taking advantage of the short generations of bacteria and the ease of manipulating their environments, we have assessed the effect of environmental variation on the reproducibility of evolution, with implications for understanding the structure of adaptive landscapes. This study extends previous work on the effects of environmental heterogeneity [13-15,19,26,28,29,61] by focusing specifically on the effect of temporal fluctuations in resource availability on population divergence. We observed that divergence among replicate populations was greater in environments with alternating resources than in environments with the same resources presented either singly or simultaneously, suggesting that epistatic interactions among mutations were stronger or more influential under the temporally fluctuating regimes. Future work will take advantage of the potential for genetic analysis and manipulation of *E. coli *to examine the molecular and physiological changes that underlie the parallel and divergent trends observed in this study.

## Conclusions

In this study, we sought to test the effect of the number (one or two) and presentation (simultaneous or fluctuating) of limiting resources on the adaptation and divergence of replicate populations of *E. coli*. The fitness of all populations increased relative to their common ancestor following 2,000 generations of evolution, indicating that all populations adapted to their selective environments. Divergence among the replicate populations evolved in each particular environment differed across regimes, with variation tending to be greater in fluctuating environments than in constant environments, including those regimes with two simultaneous resources. These results are consistent with the hypothesis that environmental heterogeneity, including temporal fluctuations, can promote population divergence.

## Methods

### Bacterial strains and propagation

The bacteria used as the ancestors in this study have been described previously [16]. Briefly, all populations were derived form a strictly asexual strain of *E. coli *B. The parental strain was unable to utilize the sugar L-arabinose (Ara-) but a mutant able to grow on this sugar (Ara+) was isolated. These strains can be distinguished by plating on tetrazolium arabinose (TA) indicator medium [16]. On this medium, Ara+ bacteria form white colonies while Ara- bacteria form red colonies. Competition assays were performed (as described below) to test whether the Ara marker had any fitness effect. The Ara marker had no significant effect on fitness in any of the environments used in our study (environments and fitness assays as described below; 2-tailed *t*-tests comparing Ara+ vs. Ara- relative fitness (r): glucose r = 1.002, *t*_18 _= 0.265, *P *= 0.794; maltose r = 1.006, *t*_16 _= 0.775, *P *= 0.500; lactose r = 0.984, *t*_16 _= 1.238, *P *= 0.221; glucose+maltose r = 0.990, *t*_15 _= 0.921, *P *= 0.372; glucose+lactose r = 1.022, *t*_15 _= 1.396, *P *= 0.183; glucose/maltose r = 0.987, *t*_15 _= 0.859, *P *= 0.404; glucose/lactose r = 0.998, *t*_15 _= 0.199, *P *= 0.845).

Bacteria were propagated under one of seven different environmental regimes, differing only in the identity and temporal presentation of the limiting resource(s). These resource regimens (and the designations used in this study) were: glucose (glu), maltose (mal), lactose (lac), glu and mal together (glu+mal), glu and mal alternating daily (glu/mal), glu and lac together (glu+lac), and glu and lac alternating daily (glu/lac). Six replicate populations, three founded by each Ara marker type, were propagated in each of the seven regimes, giving a total of 42 evolving populations. We emphasise that these populations were initially homogeneous; thus, de novo mutation was the only source of genetic variation. For the purposes of analysis and to allow comparisons across resource types, the set of four regimes containing either glu, mal, or both are referred to collectively as the glu&mal set; similarly, the set of four regimes containing glu, lac, or both are referred to as the glu&lac set. (The glu regimen is common to both sets.) The six replicate populations evolving under each regime are referred to as groups. Sugars were added to base Davis minimal (DM) media at the following concentrations to make single and mixed resource environments: glu 175 μg/ml; mal 250 μg/ml; lac 210 μg/ml; glu+mal 87.5 μg/ml and 125 μg/ml, respectively; and glu+lac 87.5 μg/ml and 105 μg/ml, respectively. These concentrations were chosen such that each environment supported nearly the same stationary-phase density of bacteria (~3.5 × 10^8 ^cfu/mL). All populations were incubated at 37°C in 1 mL × 96 well blocks (Qiagen, Valencia, CA) with shaking at ~120 rpm. Populations were propagated daily by transferring 10 μL to wells in a new block, allowing ~6.64 ( = log_2 _100) generations per day for 300 days, to give a total of ~2000 generations. Population samples were frozen every 100 generations at -80°C after addition of glycerol as a cryoprotectant.

Several precautions were taken to monitor the possibility that external or cross-contamination occurred during the propagation, allowing us to take corrective action if needed. The ancestral strain possesses several characteristic markers that were checked periodically throughout the experiment [16]. At no time were any bacteria observed that differed from the ancestral marker profile; therefore, it is unlikely that there was any external contamination. Monitoring for cross-contamination between populations was facilitated by the arrangement of the evolving populations in a checker-board pattern in the propagation block. In this arrangement, the four wells nearest to each population contained sterile medium. Observation of bacterial growth in these wells provided a sensitive means by which to detect any splashing that could contaminate adjacent wells. On several occasions such contamination was observed, and, in these cases, the experiment was restarted from the previous day's block, which had been kept overnight at 4°C. As an additional precaution, populations of opposite Ara marker types were grown adjacent to one another. Periodic plating of populations to TA indicator medium allowed us to detect possible cross-contamination. This screening found cross-contamination of one replicate mal population, and this population has been dropped from our analyses.

### Fitness assays

The fitness of each evolved population relative to the ancestor was assayed by competitions carried out in the same conditions that prevailed during the evolution experiment. In all cases, the fitness of an evolved population was compared to the ancestor bearing the opposite Ara marker in order to allow the two types to be distinguished. Before each fitness assay, the two competitors were acclimated to the competition environment by growing them separately in the same environmental conditions used in the competition. Each competitor was then diluted 200-fold into fresh DM medium supplemented with the appropriate sugar(s), and a sample immediately plated on TA agar to estimate the initial densities of the competing strains. At the end of the competition, a sample was plated on TA agar to obtain the final density of each competitor. The fitness of the evolved strain relative to the ancestor was calculated as ln(N_E_(1)/N_E_(0))/ln(N_A_(1)/N_A_(0)), where N_E_(0) and N_A_(0) represent the initial densities of the evolved and ancestral strains, respectively, and N_E_(1) and N_A_(1) represent corresponding densities at the end of the competition. Competitions lasted one day except for those performed in the fluctuating resource environments; those competitions ran for two days, such that competing strains were exposed to both sugars and both environmental transitions over the course of the assay, with the final density of each strain adjusted for the 100-fold dilution between the two days. For each group of replicate populations, fitness was measured in their own evolution environment (to assess their direct response to selection) and in the three alternative environments making up each four-regime set of the experiment (to measure their correlated responses to selection). Fitness assays were replicated four- and nine-fold to measure the direct responses after 1000 and 2000 generations, respectively, and five-fold for each correlated response.

### Statistical analyses

Analyses of variance and estimation of variance components were carried out using JMP 5.1 from the SAS Institute. In all cases, population was tested as a random factor and environmental regime was tested as a fixed factor. Student's t-tests were performed in Microsoft Excel. Significance was first assessed using α = 0.05 as the cut-off. However, we often had to interpret overall trends based on the outcome of multiple tests and comparisons. In these cases, sequential Bonferroni corrections were performed as described in table legends [62]. To reduce the influence of day-to-day variation in assay conditions, all comparisons were performed using data collected in complete blocks. Therefore, tests of among-population variation based on the direct and correlated responses of populations included only the five direct responses measured in the same experimental blocks as the correlated responses. Similarly, comparisons of direct fitness measurements at 1000 and 2000 generations used only the four 2000-generation fitness assays performed in the same experimental blocks as the 1000-generation assays.

## Authors' contributions

TC performed and analyzed the experiments. TFC and REL were responsible for the overall design and direction of the experiments. Both authors contributed substantially to writing this paper, and approved the final manuscript.
